# Anti-*Trypanosoma cruzi* Potential of Vestitol Isolated from Lyophilized Red Propolis

**DOI:** 10.3390/molecules28237812

**Published:** 2023-11-28

**Authors:** Lucas Resende Dutra Sousa, Tatiane Roquete Amparo, Gustavo Henrique Bianco de Souza, Aline Tonhela Ferraz, Kátia da Silva Fonseca, Amanda Scofield de Azevedo, Andréa Mendes do Nascimento, Ângela Leão Andrade, Janaína Brandão Seibert, Thalita Marcolan Valverde, Saulo Fehelberg Pinto Braga, Paula Melo de Abreu Vieira, Viviane Martins Rebello dos Santos

**Affiliations:** 1 Phytotechnology Laboratory, School of Pharmacy, Federal University of Ouro Preto, Campus Morro do Cruzeiro, Ouro Preto 35400-000, MG, Brazil; lucasresendedutrasousa@gmail.com (L.R.D.S.); tatianeroquete@yahoo.com.br (T.R.A.); guhbs@ufop.edu.br (G.H.B.d.S.); 2 Morphopathology Laboratory, Center for Biological Sciences Research, Federal University of Ouro Preto, Campus Morro do Cruzeiro, Ouro Preto 35400-000, MG, Brazil; aline.tonhela@aluno.ufop.edu.br (A.T.F.); katia.fonseca@gmail.com (K.d.S.F.);; 3 Department of Chemistry, Institute of Exact and Biological Sciences, Federal University of Ouro Preto, Campus Morro do Cruzeiro, Ouro Preto 35400-000, MG, Brazil; amanda.azevedo@aluno.ufop.edu.br (A.S.d.A.); andnascimento@ufop.edu.br (A.M.d.N.); angelaleao@ufop.edu.br (Â.L.A.); 4 Natural Products Laboratory, Department of Chemistry, Federal University of São Carlos, Rod. Washington Luiz, Sao Carlos 13565-905, SP, Brazil; jana_seibert@hotmail.com; 5 Department of Morphology, Institute of Biological Sciences, Federal University of Minas Gerais, Belo Horizonte 31270-901, MG, Brazil; thalitamarcolan@gmail.com; 6 Medicinal Chemistry and Bioassays Laboratory, School of Pharmacy, Federal University of Ouro Preto, Campus Morro do Cruzeiro, Ouro Preto 35400-000, MG, Brazil; saulobragaqf@gmail.com

**Keywords:** Chagas disease, cytotoxicity, isolation, membrane permeability, red propolis, *T. cruzi*, vestitol, molecular docking, farnesyl diphosphate synthase

## Abstract

Chagas disease (CD) is a worldwide public health problem, and the drugs available for its treatment have severe limitations. Red propolis is a natural extract known for its high content of phenolic compounds and for having activity against *T. cruzi*. The aim of this study was to investigate the trypanocidal potential of red propolis to isolate, identify, and indicate the mode of action of the bioactive compounds. The results revealed that the total phenolic content was 15.4 mg GAE/g, and flavonoids were 7.2 mg QE/g. The extract was fractionated through liquid–liquid partitioning, and the trypanocidal potential of the samples was evaluated using the epimastigote forms of the Y strain of *T. cruzi*. In this process, one compound was characterized by MS, ^1^H, and ^13^C NMR and identified as vestitol. Cytotoxicity was evaluated employing MRC-5 fibroblasts and H9C2 cardiomyocytes, showing cytotoxic concentrations above 15.62 μg/mL and 31.25 μg/mL, respectively. In silico analyses were applied, and the data suggested that the substance had a membrane-permeation-enhancing effect, which was confirmed through an in vitro assay. Finally, a molecular docking analysis revealed a higher affinity of vestitol with farnesyl diphosphate synthase (FPPS). The identified isoflavan appears to be a promising lead compound for further development to treat Chagas disease.

## 1. Introduction

Chagas disease (CD) was described by Carlos Chagas in 1909, and it is currently considered to be neglected by the World Health Organization [1,2]. Its etiologic agent is *T. cruzi*, a hemoflagellate protozoan that causes one of the main endemic diseases in Latin America [2,3]. This disease is endemic in 21 countries, affecting 6 million people, with an estimated 70 million people at risk of contracting the disease [4]. In addition, it is responsible for the increase in mortality in several countries and can be transmitted in different ways, thus becoming a public health problem [4,5].

Benznidazole (BZ) and nifurtimox (NF) are the only drugs available for the treatment of CD. However, they exhibit a limited efficacy and cause frequent and severe adverse effects [6,7,8]. Due to such problems, several therapeutic strategies have been designed to improve the current treatment of CD, including the use of natural products for the discovery of new active molecules, since these products can be very effective and are important sources of new drugs [9,10].

In order to develop new drugs for the CD treatment, important steps must be considered, including isolating the compound to be tested; evaluating the biological activity through in vitro tests; identifying the differences in activities toward the parasite and the host; performing an analysis of the structure–activity relationship of the isolated substance; and carrying out structural studies for the production of compounds with different chemical structures [9].

Propolis, a natural product produced by bees, comes in different colors and is widely employed in folk medicine for various diseases. Different types of propolis are available on the market in various pharmaceutical forms [11]. This product is a natural and non-toxic resin, which has been used for its antimicrobial, anti-inflammatory, anesthetic, and cariostatic properties [12,13,14]. In addition to activities already known, in recent decades, several studies have been carried out on other biological and pharmacological activities on the chemical composition and therapeutic uses of propolis [15,16,17]. In this context, propolis could be used as a natural alternative to combat neglected diseases, since it contains many substances with different potentials.

The main compounds found in different types of propolis belong to several primary classes, including phenolic compounds (phenolic acids and their esters, flavonoids such as flavones, flavonones, flavonols, dihydroflavonols, and chalcones), terpenes, steroids, aromatic aldehydes, alcohols, naphthalene, stilbenes derived from benzopyran, benzophenone, as well as derivatives of cinnamic and benzoic acids [11]. Flavonoids represent the most common and widely distributed phenolic group in red propolis and are highly associated with activity against *T. cruzi* [17,18]. In this context, the aim of this study was to investigate the total phenolics and flavonoids content in a red propolis sample in order to evaluate the trypanocidal effect of different red propolis derivatives and isolate one of its main active compounds to perform in vitro and in silico analyses. Red propolis extracts have been tested for their effectiveness against *Trypanosoma* species. However, no research has been conducted to identify the particular compound in this sample responsible for the activity against *T. cruzi*, and no studies have suggested its mode of action.

## 2. Results and Discussion

### 2.1. Total Phenolic and Flavonoid Contents

Red propolis, the second most produced and commercialized type among various Brazilian propolis varieties, stands out prominently [19,20]. The biological activities of Brazilian red propolis are strongly dependent on the concentration of active constituents, which are mainly phenolic and flavonoid compounds. In our study, the total phenol content was 15.4 ± 0.1 mg GAE/g lyophilized red propolis, and the flavonoid content was 7.2 ± 0.1 mg QE/g lyophilized red propolis. Some studies that used ethanolic-extract red propolis have reported the phenolic concentrations ranging from 7.33 to 232.00 mg GAE/g sample [21,22]. Regarding the flavonoid content, according to the studies of Righi et al. [22], the flavonoid content of red propolis collected in the Alagoas State varies from 27 to 43 mg/g sample. This difference is expected, as in addition to the influence of the sample region, the collection season and other factors can interfere [23]. Although many studies having been conducted using propolis from different origins, there are few with lyophilized red propolis. Gulçin et al. [24], while studying the lyophilized aqueous extract of propolis (LAEP), found a value of 124.3 μg GAE/g for LAEP. The content of total flavonoids in the LAEP was determined spectrophotometrically as 8.15 μg QE/g of the LAEP. The ethanolic extract investigated in this report was obtained using lyophilization and yielded fewer phenolic compounds compared to the lyophilized aqueous extract results reported by Gulçin et al. [24]. Again, it is important to note that the extraction of flavonoids and phenolic compounds can produce different results as this also depends on the polarity of the solvent used and the extraction technique employed [25].

### 2.2. Determination of Trypanocidal Activity in Epimastigote Form of Y Strain of Trypanosoma cruzi

There are various approaches to assessing anti-*T. cruzi* in vitro [3,26]. For the screening of trypanocidal activity, samples were evaluated against epimastigote forms of the Y strain of *T. cruzi*, which is considered highly pathogenic and partially resistant to conventional treatment with BZ [27,28]. Epimastigote forms are found in the digestive tract of vectors, such as triatomines, which are responsible for transmitting *T. cruzi* between vertebrate hosts [29]. In this context, the ultimate goal of such tests is to identify new drug candidates for potential development as therapies for the treatment of CD.

Table 1 presents the results obtained with different isolated compounds. In the initial isolation step, the trypanocidal activity on the epimastigote forms of the ethanolic extract (EEP-75% heated) was evaluated, revealing an IC_50_ = 12.8 μg/mL. Similar findings were reported by Dantas et al. [30], who observed a significant reduction in the epimastigotes of the same strain when assessed with ethanolic extracts of red propolis. The study further highlighted that ethanolic extracts of red propolis exhibited greater activity compared to extracts from other propolis types, underscoring the enhanced potential of red propolis [31]. In the subsequent step, two solvents, chloroform and hexane, were used to partition compounds from the most active extract. The chloroform partition displayed the highest activity, with an IC_50_ of 8.1 μg/mL (more active than the original extract), and was consequently selected for further steps. The heightened activity of the chloroform partition may be attributed to a higher content of flavonoids and aromatic acids, which are generally present in higher concentrations in red propolis, which are highly related to the inhibition of the epimastigotes and trypomastigotes of *T. cruzi* [15,17,32,33]. In the third and fourth steps, samples PV-C2 and PV-C2-4 were selected, respectively, using the same criteria as described above. In both cases, there was an observed increase in activity against the parasites. Finally, in the last step (assessment of the isolated compound), a slight increase in activity against the parasite was noted (IC_50_ = 5.2 μg/mL). The isolated compound belongs to a class of flavonoids, which is phenolic compounds considered as the primary anti-*T. cruzi* bioactives in Bulgarian propolis extracts [17,18].

Rocha et al. [34] showed the trypanocidal activity of other flavonoids derived from *Arrabidaea brachypoda* against trypomastigotes from the Y strain of *T. cruzi*. Similarly, Florencio et al. [35] observed a comparable effect of 2″,3″-dihydroochnaflavone against the same strain of *T. cruzi*, underscoring the significant potential of this compound class against the parasite. In the same context, the anti-*Trypanosoma brucei* (a flagellated protozoan species of the Trypanosomatidae family) activity of compounds isolated from Nigerian red propolis, similar to that found in Brazilian red propolis, has already been reported. In this work, one of the isolated compounds was the same as this report, vestitol, presenting IC_50_ = 8.3 μg/mL against *Trypanosoma brucei* [36]. Despite being parasites of the same genus, a notable distinction in the composition of the plasma membrane is apparent between them [37]. In particular, *T. brucei* has a comparatively high cholesterol content in its blood form when compared to other trypanosomatids [38]. Therefore, it is essential to evaluate the effect of this compound on different species. The study conducted by Tasdemir et al. [39] investigated the impact of six isoflavones on the trypomastigote forms of the Tulahuen strain of *T. cruzi* and on the blood forms of *T. brucei*. The results revealed that five of these isoflavones demonstrated superior activity against *T. brucei* compared to *T. cruzi*, while only one of them exhibited comparatively higher activity against *T. cruzi*. These findings highlight the importance of evaluating the efficacy of active compounds in relation to different trypanosomatids. Thus, for the first time, the activity of vestitol against *T. cruzi* emerges as a new contribution.

### 2.3. Nuclear Magnetic Resonance (NMR) and HRMS Analysis of Vestitol (2′,7-Dihydroxy-4′-methoxyisoflavan)

**Figure 1:**
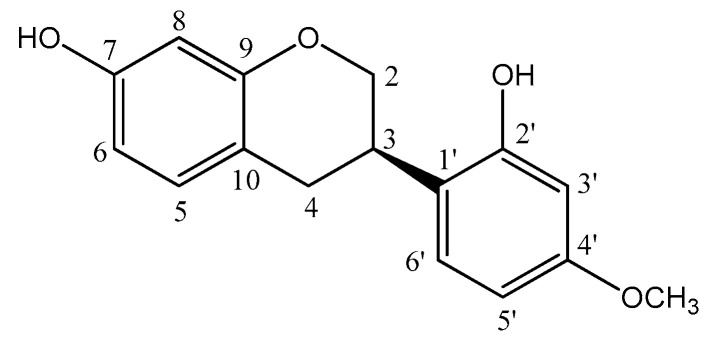
Structural representation for vestitol (**1**) isolated from lyophilized red propolis.

The NMR data of compound **1** was compared to data found in the literature [40,41] and confirmed the assignment of compound **1** as vestitol (2′,7-dihydroxy-4′-methoxyisoflavan) (Figure 1). The isoflavan was isolated from the chloroformic partition (as a white compound) and displayed characteristic ^1^H and ^13^C NMR spectra (Figures S1–S3, Supplementary Material). The aromatic region of the ^1^H NMR spectrum of the isoflavan contained three signals at δ 6.35 (d, 1H, *J* = 2.5 Hz, H-8), 6.39 (dd, 1H, *J* = 8.0, 2.5 Hz, H-6), and 6.94 (d, 1H, *J* = 8.0 Hz, H-5), suggesting the presence of a monosubstituted ring A. Three signals at δ 6.36 (d, 1H, *J* = 2.5 Hz, H-3′), 6.47 (dd, 1H, *J* = 8.5, 2.5 Hz, H-5′), and 7.01 (d, 1H, *J* = 8.5 Hz, H-6′), were consistent with the signals of a 2′,4′-disubstituted ring B. The ^1^H NMR spectrum also showed signals at δ 4.03 (t, 1H, *J* = 10.5 Hz, H-2ax), 4.32 (dd, 1H, *J* = 10.5, 2.5 Hz, H-2eq), 3.52–3.47 (m, 1H, H-3ax), 2.99 (dd, 1H, *J* = 15.0, 10.5 Hz, H-4ax), and 2.90 (dd, 1H, *J* = 15.0, 5.5, H-4eq), assigned to the heterocyclic protons of isoflavan moiety and also confirmed through signals in the ^13^C NMR spectrum (δ 69.9 for C-2, δ 31.7 for C-3, and δ 30.4 for C-4). The ^13^C NMR spectrum showed 16 spectral lines, 4 of which were found in the characteristic region of sp^2^ carbon and 4 corresponding to oxygenated carbons at δ 159.3 (C-4′), 155.1 (C-7), 154.9 (C-9), and 154.4 (C-2′), in addition to a sign relative to a methoxyl group at δ 55.3 (OCH_3_-4′).

^1^H NMR (500 MHz, CDCl_3_) δ 2.90 (dd, 1H, *J* = 15.0, 5.5, H-4eq); 2.99 (dd, 1H, *J* = 15.0, 10.5, H-4ax), 3.52–3.47 (m, 1H, H-3ax), 3.76 (s, 3H, OCH_3_-4′), 4.32 (dd, 1H, *J* = 10.5, 2.5, H-2eq), 4.03 (t, 1H, *J* = 10.5, H-2ax), 6.35 (d, 1H, *J* = 2.5, H-8), 6.36 (d, 1H, *J* = 2.5, H-3′), 6.39 (dd, 1H, *J* = 8.0, 2.5, H-6), 6.47 (dd, 1H, *J* = 8.5, 2.5, H-5′), 6.94 (d, 1H, *J* = 8.0, H-5), 7.01 (d, 1H, *J* = 8.5, H-6′); ^13C^ NMR (125 MHz, CDCl_3_) δ 30.3 (CH_2_-4); 31.7 (CH-3), 55.3 (OCH_3_-4′), 69.9 (CH_2_-2), 102.1 (CH-3′), 103.2 (CH-8), 105.9 (CH-5′), 107.9 (CH-6),114.6 (C-10), 119.9 (C-1′), 128.1 (CH-6′), 130.4 (CH-5), 154.4 (C-2′), 154.9 (C-9), 155.1 (C-7), 159.3 (C-4′).

The molecular formula C_16_H_16_O_4_ of the vestitol was deduced from the *pseudo*-molecular-ion peak at *m*/*z* 273.1671 ([M + H]^+^; calc. 273.3053) in the positive-ion-mode HRMS (ESI-TOF) (Figure S4, Supplementary Material).

### 2.4. Cytotoxicity Assay

Evaluating the cell viability of isolated compounds is essential to understand possible harmful effects and establish safe dosages for the intended application [42]. In this study, ISO 10993-5:2009 was employed as a guideline, which suggests that samples are considered cytotoxic when viability is below 70% [43].

MRC-5 cells are commonly used to evaluate the safety profile of both natural and synthetic compounds [44,45]. This cell line is often featured in scientific investigations and laboratory experiments that aim to scrutinize various cellular processes, responses to external stimuli, and intricate cellular interactions. One of its notable features is its rapid and efficient proliferation [46]. Furthermore, due to the heart’s importance as a target for *T. cruzi*, it is important to assess the survival of H9C2 cells when exposed to possible bioactive compounds against this parasite [47].

**Figure 2:**
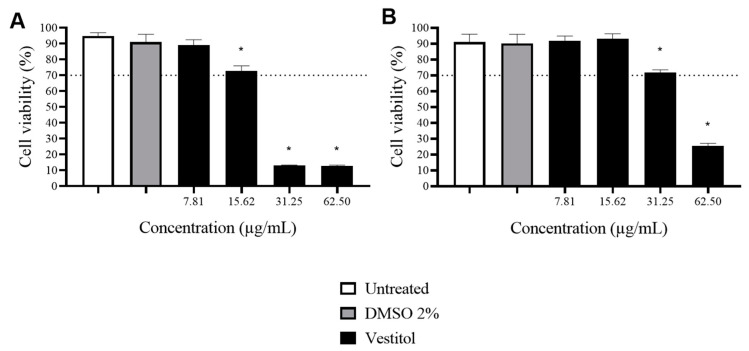
Cell viability in MRC-5 fibroblasts (**A**) and in H9C2 cardiomyocytes (**B**) exposed to different concentrations of vestitol (7.81 to 62.50 μg/mL). Results represent the mean ± SD of triplicates of the experiments; (*) denotes a significant difference compared to the untreated group (*p* ≤ 0.05), as determined using one-way ANOVA followed by a Dunnett’s post-test.

Vestitol exhibited no cytotoxic effects at concentrations of up to 15.62 μg/mL for MRC-5 fibroblasts (Figure 2A) and up to 31.25 μg/mL for H9C2 cardiomyocytes (Figure 2B). This is possibly advantageous since these concentrations are three and six times higher than the IC_50_ value of this compound against *T. cruzi* (5.2 μg/mL), respectively.

The cytotoxic activity of vestitol has been exhibited in various tumor cell lines with the aim of identifying potential antitumor drugs for future use [48,49]. However, there is a scarcity of information on its cytotoxicity in non-tumorous cell lines. To the best of our knowledge, in addition to our results, only the work by Bueno-Silva et al. [50] has addressed this aspect. In this project, the anti-inflammatory activity of vestitol isolated from Brazilian red propolis using RAW 264.7 macrophages was evaluated. For such an evaluation, it was necessary to evaluate the viability of these cells against this compound. In addition to the cells remaining viable at concentrations between 0.37 and 0.59 μM, vestitol was able to reduce the production of nitric oxide (NO) by 83% at 0.55 μM, which would be interesting in possible new treatments for CD, since the production of NO produced in the presence of *T. cruzi* can lead to the development of severe forms of CD [51].

### 2.5. In Silico and In Vitro Investigations of the Mechanism of Action

In silico studies are important tools used to help direct in vitro tests in researching mechanisms of action [52]. The potential biological properties of the isolated isoflavan related to anti-*T. cruzi* activity were investigated using PASS online. This tool enables the prediction of the biological activities of a chemical compound based on its molecular structure, with the results expressed as probability to be active (Pa) and probability to be inactive (Pi).

The results of vestitol that presented a Pa value higher than a Pi value were selected, and membrane permeability enhancer (Pa-Pi 0.263) and cholesterol synthesis inhibitor (Pa-Pi 0.190) parameters were suggested as potential biological effects. Although ergosterol is the constituent of the *T. cruzi* membrane, many enzymes are also common for cholesterol production. Therefore, in silico results suggest that an action on a parasite plasma membrane would be the probable mechanism of action of vestitol. A flow cytometry assay using a propidium iodide (PI) fluorescent marker was used to evaluate this effect in vitro.

**Figure 3:**
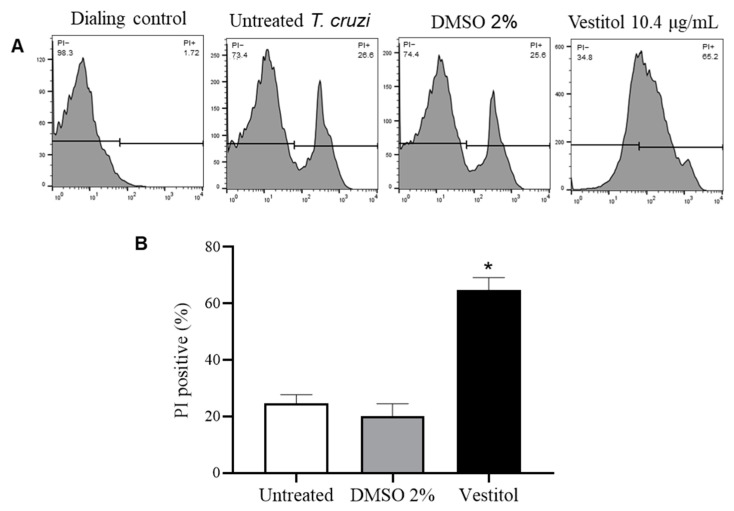
Flow cytometry analysis of *Trypanosoma cruzi* membrane permeability of the controls (untreated cells and DMSO 2%) and after treatment with vestitol. (**A**) Histograms; (**B**) Percentage of damaged *Trypanosoma cruzi* cells stained with propidium iodide (PI). (*) denotes a significant difference compared to the untreated cells (*p* ≤ 0.05), as determined using one-way ANOVA followed by a Dunnett’s post-test.

An increase in PI labeled *T. cruzi* cells was observed after treatment with vestitol (Figure 3A), and this difference was statistically significant compared to the untreated *T. cruzi* (Figure 3B). This in vitro result confirms that the mode of action of vestitol involves the loss of membrane integrity, since the PI is a membrane-impermeable agent that binds to the nucleic acids when the membrane is compromised. DMSO 2%, used to dissolve vestitol in the medium, did not cause an increase in PI labeled in relation to untreated cells, showing that the preparation of isoflavan for the test did not affect the results (Figure 3).

This effect on a parasite plasma membrane may be related to ergosterol biosynthesis inhibitors (EBI) that cause an imbalance in the fluidity of this membrane [53]. Studies with extracts of Bulgarian propolis, rich in flavonoids, suggest that the inhibition of the epimastigote forms of *T. cruzi* is associated with the disruption of lipid biosynthesis, causing alterations in the biophysical properties of the plasma membrane of the treated parasite and, consequently, its lysis [17,18]. Furthermore, according to Tagousop [54], the mode of action of three flavonoids isolated from *Graptophyllum grandulosum* is associated with cell lysis and increased membrane permeability. Thus, EBI are promising candidates as anti-*T. cruzi* agents, since the parasite needs several sterols for its survival, and these cannot be replaced with the cholesterol of the host [37].

Among the enzymes involved in an ergosterol biosynthesis considered as anti-*T-cruzi* potential targets, farnesyl diphosphate synthase (FPPS), lanosterol C-14 demetilase (CYP51), and squalene synthase (SQS) are highlighted due to their antitrypanosomal results already reported and their well established crystal structures for *T. cruzi* [37,55,56,57]. Thus, these enzymes were included in the molecular docking analysis.

To validate the docking procedure, re-docking and superimposition were used. The crystallographic ligands were re-docked into the active site of each target using PyRx. Subsequently, the re-docked complexes were superimposed onto the native co-crystallized enzymes from the Protein Data Bank (PDB) using Biovia Discovery Studio software v.21.1. Notably, low root mean square deviation (RMSD) values were observed: 1.1662 Å for CYP51, 0.3685 Å for SQS, and 1.3001 Å for FPPS (Figure S5, Supplementary Material). The re-docked complex was found to interact with similar amino acid residues when compared to the native co-crystallized complexes (Figure S5, Supplementary Material). These results suggest that the docking protocol was efficient and valid [58].

Vestitol exhibited the best affinity to FPPS among the analyzed targets (Table 2). This enzyme participates in the isoprenoid pathway for sterols biosynthesis, such as ergosterol and cholesterol. They catalyze the successive condensation of isopentenyl diphosphate (IPP) and dimethylallyl diphosphate (DMAPP) to generate geranyl diphosphate (GPP), then farnesyl diphosphate (FPP), which is the branching point in the mevalonate pathway [55,59]. Consequently, an FPPS inhibition leads to a decrease in the production of isoprenoid compounds such as sterols [37,59]. Thus, FPPS is considered a possible target for anti-*T. cruzi* drugs since ergosterol is an essential membrane sterol in trypanosomatid parasites [37].

The binding energy values of vestitol indicate stronger affinity with FPPS than the crystallographic ligand PB6 (Table 2). PB6 (1-(2,2-bis-phosphono-ethyl)-3-butyl-pyridinium) is from the class of nitrogen-containing bisphosphonates, considered promising therapeutic agents against trypanosomal infections, acting as analogues of isoprenoid diphosphates, and inhibiting FPPS [55].

**Figure 4:**
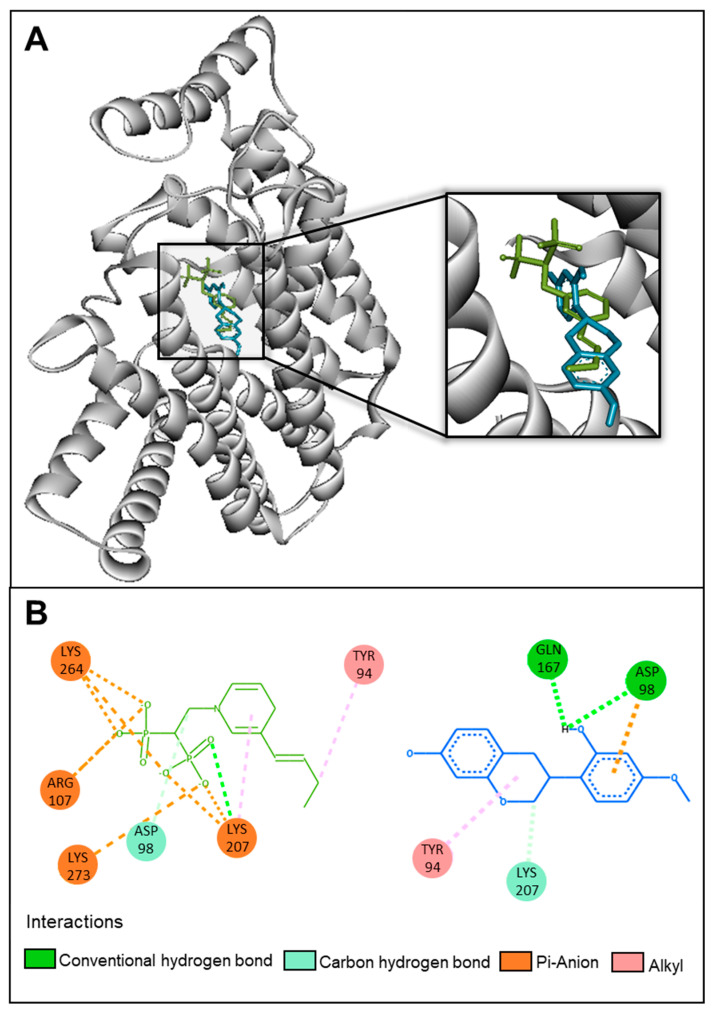
A 3D diagram (**A**) and 2D interactions diagram (**B**) showing the binding site of vestitol (blue) and PB6 (green) with farnesyl diphosphate synthase (FPPS).

Docking results indicated that vestitol bound to the same site as PB6 (Figure 4A); thus, it could also inhibit the FPPS activity. The isoflavan interaction includes hydrogen bonds with the FPPS residues Gln167, Asp98, and Lys207, as well as hydrophobic bonds with Tyr94. Among these interactions, the majority are with similar amino acid residues compared to the crystallographic ligand PB6 (Asp98, Lys207, and Tyr94) (Figure 4B).

## 3. Material and Methods

### 3.1. General Experimental Procedures

Solvents and reagents were purchased from Synth (Rio de Janeiro, Brazil), Sigma Aldrich Chemical Co. (Burlington, MA, USA), and Vetec (Rio de Janeiro, Brazil). Polyamide CC6 (Macherey-Nagel, code 81561) and Sephadex LH-20 were used in column chromatography (CC). Preparative thin-layer chromatography (TLC) was performed on silica gel GF254 (Merck No. 1.07730).

### 3.2. Red Propolis Samples

The lyophilized red propolis samples were bought in Pharma Néctar, in the Alagoas State, located in the Northeastern Region of Brazil.

### 3.3. The Ethanolic Extract Preparation of Red Propolis (EEP 75% Heated)

The lyophilized red propolis sample (20 g) was extracted using 75% ethanol (Neon), 15 mL of ethanol for each 2 g in a water bath at 70 °C for 30 min. After that, the sample was filtered on filter paper, 100 mL of ethanol (75%) was added to the residue, and another alcoholic extraction was performed. The choice of 75% ethanol as the extraction solvent and the temperature of 70 °C adhered to a previously established methodology [60]. Throughout the extraction, the system was open and subjected to shaking. The resulting solution was dried, yielding 5.17 g of the ethanolic crude extract. This crude extract was then utilized for evaluating trypanocidal activity and subjected to a liquid–liquid partition to isolate the bioactive compound.

### 3.4. Determination of Total Phenolics Content

The total content of phenolic compounds was determined by using Folin–Ciocalteu phenol reagent with slight modifications [61,62]. Briefly, 13.3 mg of the sample was dissolved in 50 mL of absolute ethanol. Subsequently, 800 μL of the ethanolic extract was transferred to test tubes, and 600 μL of distilled water and 100 μL of Folin–Ciocalteu reagent were added, followed by homogenization for 1 min. Afterward, 400 μL of sodium carbonate solution 15% (*w*/*v*) was added. A new homogenization was performed for 30 s, and an additional 100 μL of water was added. The test tubes were kept in the dark for 120 min and then homogenized. The absorbance was recorded at a wavelength of 725 nm against a blank. The blank, prepared in the same manner, included 800 μL of ethanol instead of the ethanolic extract. The disparity between the sample and the blank represented the total phenolics content.

To quantify these extracts, a calibration curve was generated by analyzing various concentrations of gallic acid ranging from 0.0018 to 0.018 mg/mL. The absorbance data were utilized to establish the calibration equation: y = 108.44x + 0.0551 (r^2^ = 0.9981). Results were expressed in terms of milligrams of gallic acid equivalent (GAE) per gram of lyophilized red propolis weight.

### 3.5. Determination of Total Flavonoids Content

The total flavonoids content was determined using the colorimetric method with aluminum chloride (AlCl_3_) according to the protocol described by Dowd et al. [63]. A total of 1500 μL of extract (13.3 mg/50 mL in absolute ethanol) was transferred to a test tube, and 1500 μL of a 20 mg/mL ethanolic solution of aluminum chloride (AlCl_3_) was added. The samples were homogenized and allowed to stand in darkness for 10 min against a blank. The blank, prepared in a similar manner, incorporated 1500 μL of ethanol instead of the AlCl_3_ solution. The spectrophotometer was set at a wavelength of 420 nm, and the absorbance reading was measured. The calibration curve was constructed from different concentrations of quercetin varying from 0.05 to 0.7 mg/100 mL, and its data on absorbances was based on the calibration equation y = 0.2846x + 0.0044 (r^2^ = 0.9979). The results were expressed in terms of milligrams of quercetin (QE) per g of lyophilized red propolis weight.

### 3.6. Liquid–Liquid Partition with EEP-75% Heated

An ethanolic extract was subjected to the liquid–liquid partition. For this, the ethanolic extract was dissolved in water: absolute ethanol (Synth) 7:3, and 100 mL of hexane was added. The procedure was repeated again with 100 mL of *n*-hexane. An additional partition with chloroform (100 mL, twice) was performed. The resulting organic layer was evaporated to produce the hexanic (0.16 g) and chloroformic (3.39 g) partitions. The resulting partitions were used for the assessment of the trypanocidal activity.

### 3.7. Isolation of Active Compound

The chloroformic partition was selected for chemical studies according to the results of the biological activity (partition that exhibited the best value of in vitro trypanocidal activity). The chloroformic partition (3.39 g) was fractionated on reversed-phase polyamide using water and ethanol gradients, as well as ethanol and ethyl acetate gradients, to yield nine fractions (PV-C1 to PV-C9) based on TLC. Fraction PV-C2 (2.27 g), which showed the best value of in vitro trypanocidal activity, was rechromatographed on a Sephadex LH-20 column using water and methanol gradients, resulting in seven sub-fractions (PV-C2-1 to PV-C2-7). The bioactive sub-fraction PV-C2-4 (41.1 mg) was separated using preparative TLC (*n*-hexane–ethyl acetate 1:1) to yield white compound **1** (9.3 mg).

### 3.8. Determination of Trypanocidal Activity in Epimastigotes Forms of the Y Strain Trypanosoma cruzi

The trypanocidal assays were performed in five steps (according to isolation). The epimastigotes of the Y strain from *T. cruzi* were obtained in the exponential growth phase. Then, they were washed twice with a LIT medium in sterile PBS at pH 7.2 and centrifuged at 1500 rpm for 10 min at 4 °C. The number of parasites was determined by counting in a Neubauer chamber. Afterward, the parasites were suspended in a LIT medium, further supplemented with 10% Fetal Bovine Serum inactivated at 56 °C, and the concentration of the epimastigotes was adjusted to 5 × 10^6^ epimastigotes/μL. To perform a sample screening, 3 mg of the sample was dissolved in 120 µL of DMSO, and 1080 µL of the LIT medium was added. This resulted in a sample concentration of 2500 µg/mL with 10% DMSO. The other solutions were obtained by diluting serially 1:1 in the LIT medium containing 10% DMSO. From these solutions, 200 µL was transferred to 48-well plates that contained 800 µL of the LIT medium with *T. cruzi* to achieve the final concentrations of 3.90, 7.81, 15.62, 31.25, 62.50, 125.00, 250.00, and 500.00 μg/mL, while keeping the 2% DMSO. The parasites incubated in the absence of the test sample and in the presence of 2% DMSO were used as a negative control and benznidazole (3.90 to 500.00 μg/mL) as a positive control. After a 72-h incubation period, the activity was determined by counting in a Neubauer chamber. The IC_50_ on *T. cruzi* was determined using a linear curve interpolation. The tests were performed in triplicate and the results expressed as mean ± standard deviation.

### 3.9. Nuclear Magnetic Resonance (NMR) and HRMS Analyses of Vestitol

^1^H and ^13^C NMR spectra (1D experiments) were recorded on Bruker DRX 500 spectrometer (500 MHz for ^1^H and 125 for ^13^C). CDCl_3_ was used as a solvent, and TMS as an internal standard. Chemical shifts were reported in ppm and coupling constants (J values) in Hz. A high resolution mass spectrum (HRMS) was acquired using an LCMS-Q-ORBITRAP (Thermo Scientific, Fair Lawn, NJ, USA) mass spectrometer operating in a positive-ion mode HRMS (ESI-TOF), and the vestitol was solubilized in methanol and trifluoroacetic acid (0.1%) because it was not soluble in acetonitrile. In this way, vestitol was solubilized and protonated. The high-performance liquid chromatography (HPLC) was bypassed, and the vestitol was directly injected into the mass spectrometer.

### 3.10. Cytotoxicity Assay

For the evaluation of cytotoxicity in human cells, MRC-5 human fibroblasts and H9C2 rat cardiomyocytes cultivated in an RPMI 1640 medium (Sigma^®^) were distributed in a 96-well microtiter plate (density of 5 × 10^4^ cells) and subsequently incubated at 37 °C with 5% of CO_2_ for 24 h. Then, 1 mg of vestitol was dissolved in 320 µL of DMSO and added to 7680 µL of the RPMI medium. This resulted in a sample concentration of 125 µg/mL with 4% DMSO. Subsequently, 200 µL was transferred to higher-concentration wells. In the remaining wells, 100 µL of the RPMI medium was added for 1:1 serial dilution, starting with the first concentration. Then, 100 µL of the RPMI medium was added to all wells to obtain the final concentrations ranging from 7.81 to 62.50 µg/mL (containing a maximum of 2% DMSO). The cells incubated in the absence of the test sample and in the presence of 2% DMSO were used as a negative control. After a 72 h incubation period, cell viability was evaluated using the sulforhodamine B assay (SRB) [64]. The medium was removed, and cells were fixed with cold 20% trichloroacetic acid for 1 h at 4 °C. The microtiter plate was washed with distilled water and dried. Thereafter, fixed cells were stained for 30 min with 0.1% SRB dissolved in 1% acetic acid. The plate was washed again with 1% acetic acid and allowed to dry, and 200 µL of 10 mM TRIS buffer (pH 10.5) was added for stain solubilization at room temperature for ~30 min. Samples absorbance was read in the spectrophotometer (490 nm). The tests were performed in triplicate and the results expressed as mean ± standard deviation of the percentage of cell viability using the GraphPad Prism 8.0.1 software.

### 3.11. Prediction of the Biological Activity Spectra

The isoflavan isolated from the lyophilized red propolis sample was subjected to the prediction of the biological activity spectra analysis in order to indicate its trypanocidal mode of action. Biological effects related to activity against *T. cruzi* (membrane permeability enhancer, cholesterol synthesis inhibitor, and DNA synthesis inhibitor) were analyzed using the Prediction of Activity Spectra for Substances 2023 (PASS online) program. The results were expressed by the difference in the probabilities of the compound to be active (Pa) and inactive (Pi) [52].

### 3.12. In Vitro Cytoplasmic Membrane Permeability Assay

Flow cytometry using propidium iodide (PI) as a marker was used to evaluate the cell membrane permeability changes [52]. A total of 40,000 epimastigotes in the LIT medium were added to each well of a 96-well microplate. This procedure was followed by the addition of 100 μL of treatment consisting of 2 × IC_50_ of isoflavan dissolved in LIT with 2% DMSO. Only the medium or 2% DMSO were used as control. After incubation for 72 h, the samples were transferred to cytometry tubes, centrifuged, and washed with phosphate-buffered saline. Cells were resuspended in 200 μL of the same buffer and incubated with 1 μL of PI solution (1 mg/mL) for 30 min protected from light. The dialing control was prepared in the same way as the negative control but without a PI solution. Flow cytometry analyses were conducted using BD FACS Calibur (Becton Dickinson Bioscience, San Diego, CA, USA), and PI was excited using a blue laser (438 nm) and analyzed by the FL2 channel. The data were analyzed using the software Flow Jo v.10.

### 3.13. Molecular Docking

The molecular docking analysis was carried out using the AutoDock Vina tool on the PyRx software v.0.8 [65]. Crystal structures of *T. cruzi* CYP51 (PDB ID 4H6O), FPPS (PDB ID 3ICZ), and SQS (PDB ID 3WCC) were obtained from the Protein Data Bank (PDB). Initially, the protein structure was prepared for docking by removing all water molecules and bound ligands from protein using Biovia Discovery Studio software v.21.1 (San Diego, CA, USA). Three-dimensional structures of the compounds were obtained from the PubChem database. The ligands were prepared using energy minimization and converted to the AutoDock Ligand format (PDBQT) using the Open Babel module in the PyRx tool. A grid box of at least X: 10.0131, Y: 15.0256, Z: 151.1323 Å was defined to cover the active binding site: center X: 2.8664, Y: 25.3701, Z: 15.7753 for CYP51; X: 9.7558, Y:15.0155, Z: 151.1325 for FPPS; and X: 80.1552, Y: 7.3557, Z: 41.2828 for SQS. The AutoDock Vina algorithm was used to calculate the binding energies between the targets and the compounds using the PyRx docking tool, and the binding energies were expressed in kcal/mol.

Aiming to also validate the parameters of the docking approach, the crystallized ligands (PB6 for FPPS, (1-(3-(4-chloro-3,5-dimethylphenoxy)benzyl)-1*H*-imidazole for CYP51 and E5700 for SQS) were re-docked into the binding pocket. The docked complex was superimposed onto the X-ray-resolved crystal bearing the co-crystalized ligand to compute the root mean square deviation (RMSD) value using Biovia Discovery Studio software v.21.1.

### 3.14. Statistical Analysis

The cell viability (MRC-5 and H9C2 cells) and flow cytometry results of the *Trypanosoma cruzi* membrane permeability were analyzed using one-way ANOVA followed by Dunnett’s post-test. Results were reported as mean ± standard deviation, and the GraphPad Prism 8.0.1 software was utilized. Differences were considered significant when *p* ≤ 0.05.

## 4. Conclusions

This investigation elucidated the stages involved in isolating one of the primary active compounds within the ethanolic extract of Brazilian lyophilized red propolis, targeting *T. cruzi*. The isolated compound, identified as vestitol, demonstrated in vitro effectiveness against *T. cruzi* epimastigote forms and a safety profile in relation to MRC-5 and H9C2 cells. In silico results suggest that the substance has a membrane-permeation-enhancing effect, including the interaction of vestitol with FPPS, an enzyme involved in ergosterol biosynthesis, which was confirmed through an in vitro assay. Finally, a molecular docking analysis revealed a higher affinity of vestitol with farnesyl diphosphate synthase (FPPS). These outcomes corresponded with those garnered from in vitro analyses, which confirmed that the mode of action of vestitol involves loss of membrane integrity. This discovery introduces a novel molecule with promising anti-*T. cruzi* properties, bringing us closer to the development of a potential drug for Chagas disease chemotherapy.

## Figures and Tables

**Table 1:** Trypanocidal activity on epimastigote forms of *T. cruzi* (Y strain) (IC_50_).

Samples	Isolation Step	IC_50_ (μg/mL)
EEP 75% heated	1st	12.8 (±0.2)
Chloroformic partition—EEP 75% heated	2nd	8.1 (±0.3)
Hexanic partition—EEP 75% heated	2nd	31.3 (±0.4)
PV-C1	3rd	24.4 (±0.3)
PV-C2	3rd	7.2 (±0.3)
PV-C3	3rd	15.9 (±0.5)
PV-C4	3rd	9.6 (±1.3)
PV-C5	3rd	34.5 (±0.4)
PV-C6	3rd	25.8 (±0.6)
PV-C7	3rd	24.9 (±0.3)
PV-C8	3rd	22.9 (±2.6)
PV-C9	3rd	45.3 (±0.5)
PV-C2-1	4th	30.5 (±0.4)
PV-C2-2	4th	35.8 (±0.3)
PV-C2-3	4th	9.0 (±0.3)
PV-C2-4	4th	5.9 (±0.2)
PV-C2-5	4th	8.1 (±0.4)
PV-C2-6	4th	37.4 (±0.3)
PV-C2-7	4th	35.0 (±0.2)
Compound **1**	5th	5.2 (±0.2)
Benznidazole	5th	4.3 (±0.3)

IC_50_: inhibitory concentration for 50% of epimastigote form; results represent the mean ± SD of triplicates of the experiments.

**Table 2:** Molecular docking results between isoflavan from red propolis and protein targets related to *T. cruzi.*

Binding Energy (kcal/mol)		
Target	Isoflavan	Crystallographic Ligand
Lanosterol C-14 demetilase (CYP51) ^a^	−7.7	−8.0
Squalene synthase (SQS) ^b^	−7.8	−11.2
Farnesyl diphosphate synthase (FPPS) ^c^	−9.3	−8.7

Crystallographic ligand: ^a^ 1-(3-(4-Chloro-3,5-dimethylphenoxy)benzyl)-1*H*-imidazole [48]; ^b^ E5700 [49]; ^c^ PB6 [47].

## Data Availability

All data generated or analyzed during this study are included in this published article.

## Supplementary Materials

The following supporting information can be downloaded at: https://www.mdpi.com/article/10.3390/molecules28237812/s1, Figure S1: ^1^H RMN (500 MHz) spectrum of the vestitol in CDCl_3_. Figure S2: ^13^C RMN (125 MHz) spectrum of the vestitol in CDCl_3_. Figure S3: ^13^C RMN, DEPT 135º (125 MHz) spectrum of the vestitol in CDCl_3_. Figure S4: High resolution mass spectrum of the vestitol in the positive ion mode. Figure S5: Superimposition of re-docked and 2D molecular interaction analysis for CYP51 (A, B and C), squalene synthase (D, E and F) and farnesyl synthase (G, H and I). Co-crystallized ligand (green) and the re-docked ligand (blue). Red cycle: similar residues comparing co-crystallized ligand and the re-docked ligand.
