# Supplementary material for: Anti-Trypanosoma cruzi Potential of Vestitol Isolated from Lyophilized Red Propolis
Source: Molecules. 2023 Nov 28;28(23):7812. doi: 10.3390/molecules28237812 (PMC10708512; doi:10.3390/molecules28237812)
Supplement: Supplementary file 1 [file molecules-28-07812-s001.zip › molecules-2611859-supplementary.pdf]

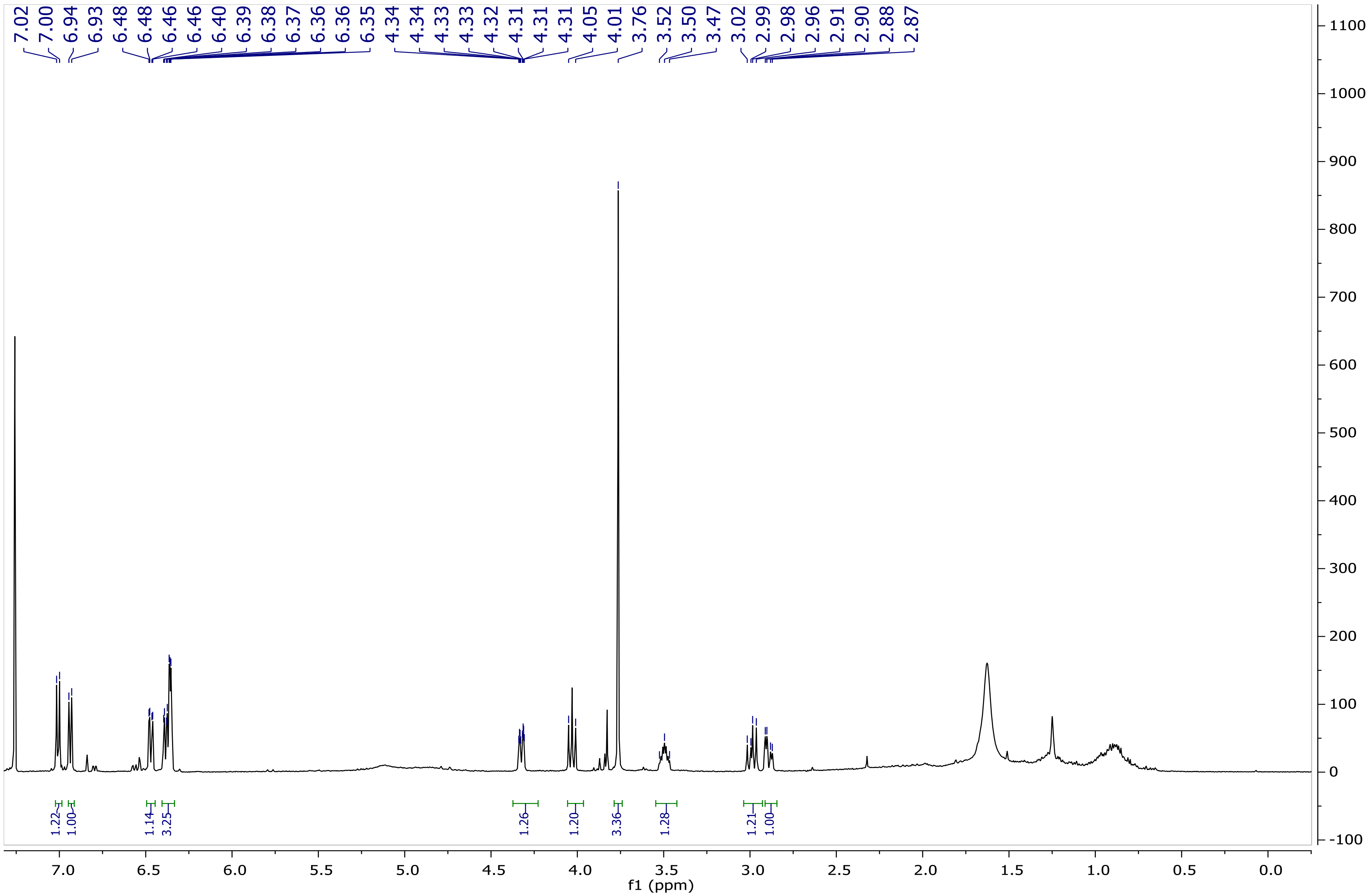

Figure S1: ^{1}H RMN (500 MHz) spectrum of the vestitol in $CDCl_3$

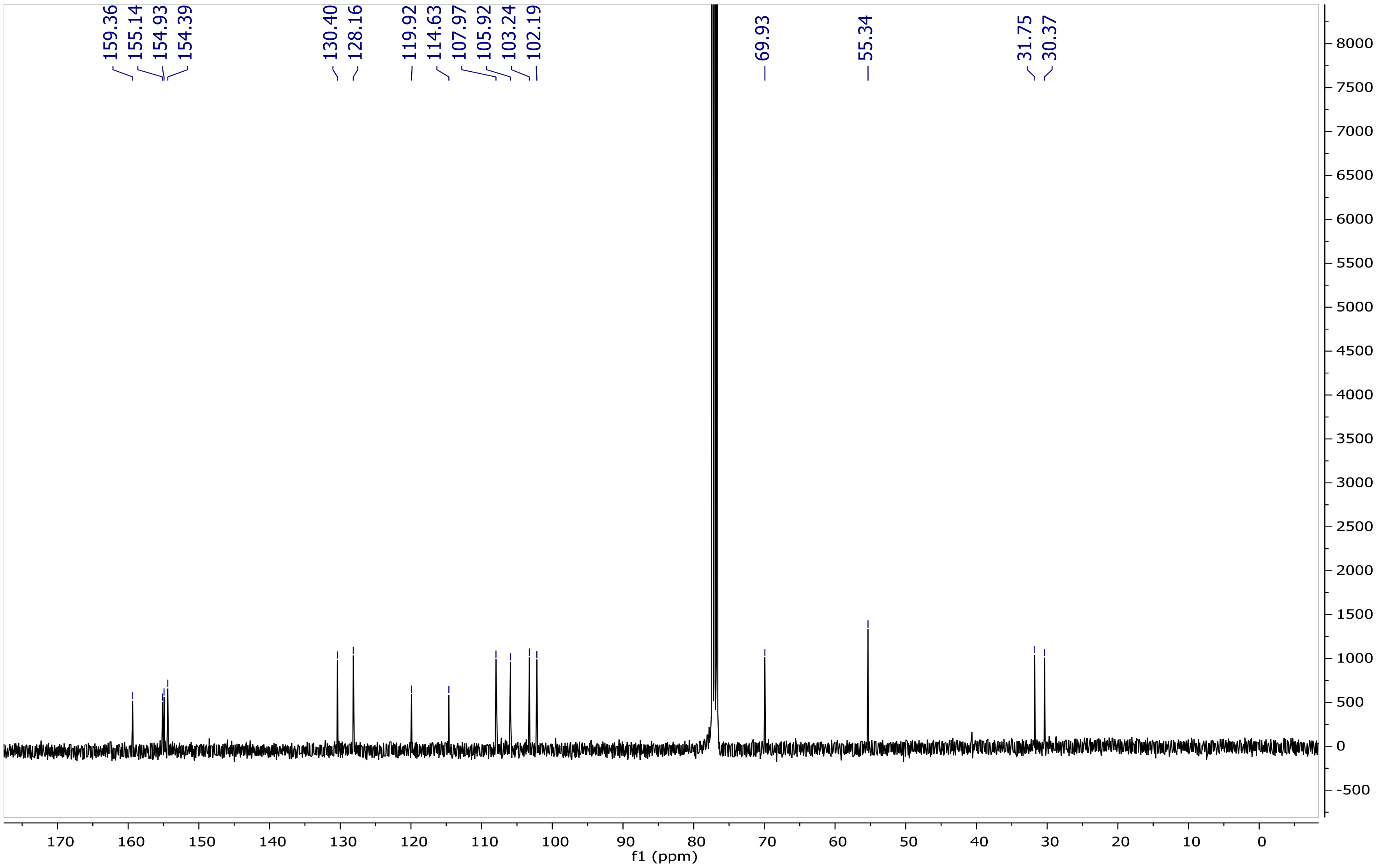

Figure S2: ^{13}C RMN (125 MHz) spectrum of the vestitol in $CDCl_3$

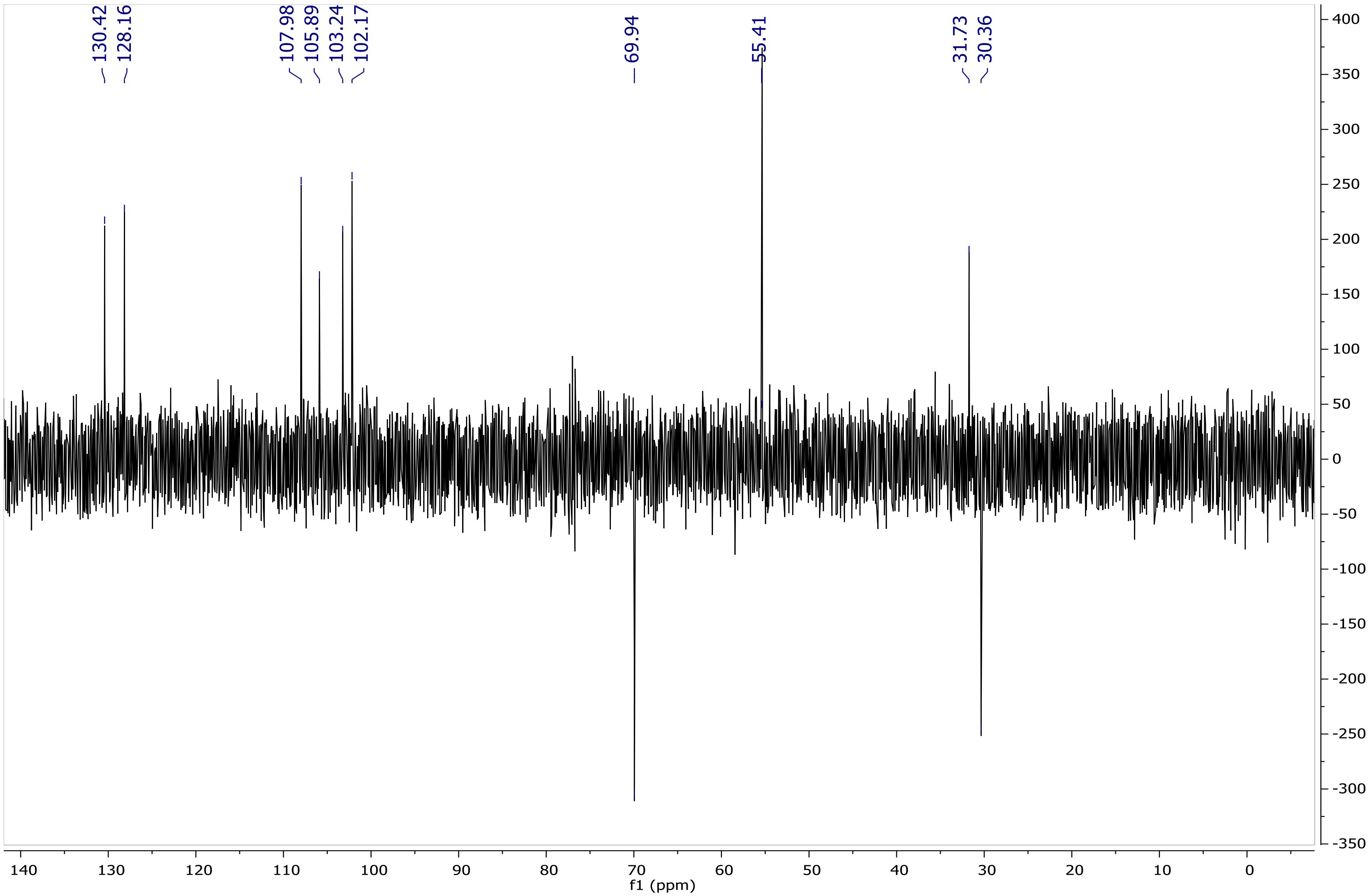

Figure S3: ^{13}C RMN, DEPT 135º (125 MHz) spectrum of the vestitol in $CDCl_3$

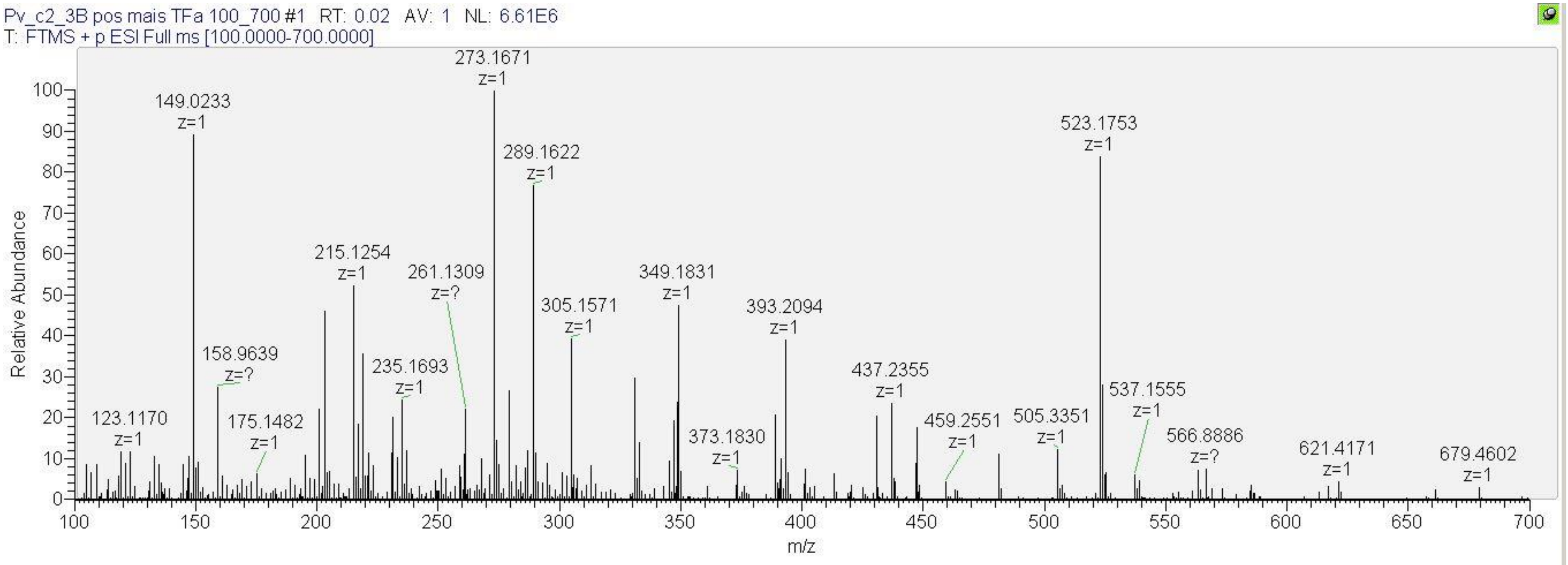

Figure S4: High resolution mass spectrum of the vestitol in the positive ion mode

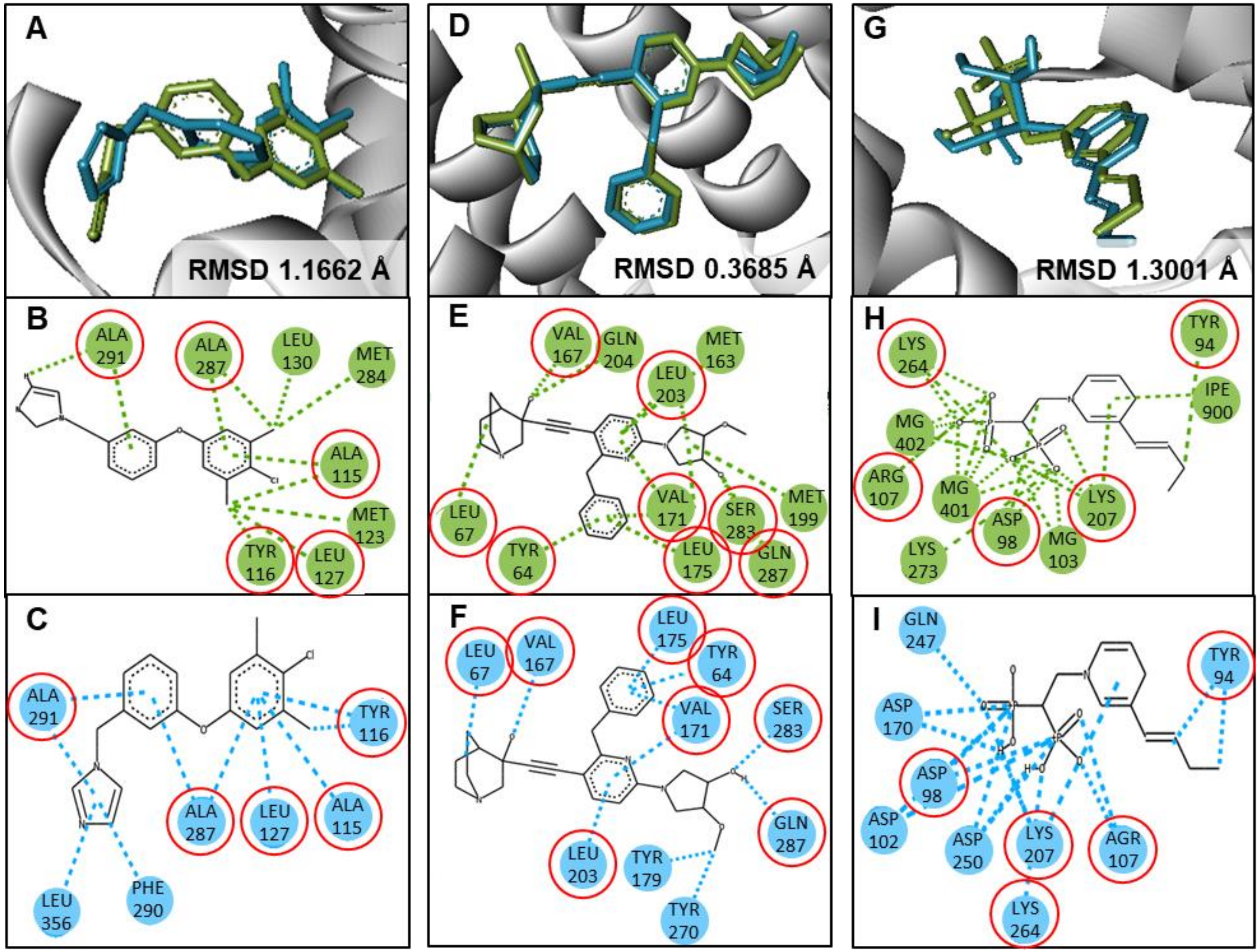

Figure S5: Superimposition of re-docked and 2D molecular interaction analysis for CYP51 (**A**, **B** and **C**), squalene synthase (**D**, **E** and **F**) and farnesyl synthase (**G**, **H** and **I**). Co-crystallized ligand (green) and the re-docked ligand (blue). Red cycle: similar residues comparing co-crystallized ligand and the re-docked ligand.
